# Self-Powered Diaper Sensor with Wireless Transmitter Powered by Paper-Based Biofuel Cell with Urine Glucose as Fuel

**DOI:** 10.1021/acssensors.1c01266

**Published:** 2021-07-15

**Authors:** Isao Shitanda, Yuki Fujimura, Tatsuya Takarada, Ryo Suzuki, Tatsuo Aikawa, Masayuki Itagaki, Seiya Tsujimura

**Affiliations:** † Department of Pure and Applied Chemistry, Faculty of Science and Technology, Tokyo University of Science, 2641, Yamazaki, Noda, Chiba 278-8510, Japan; ‡ Research Institute for Science and Technology, Tokyo University of Science, 2641 Yamazaki, Noda, Chiba 278-8510, Japan; § Division of Material Science, Faculty of Pure and Applied Science, University of Tsukuba, 1-1-1, Tennodai, Tsukuba, Ibaraki 305-5358, Japan

**Keywords:** bioanode, biocathode, body fluids, electrodes, postprandial hyperglycemia, wearable device

## Abstract

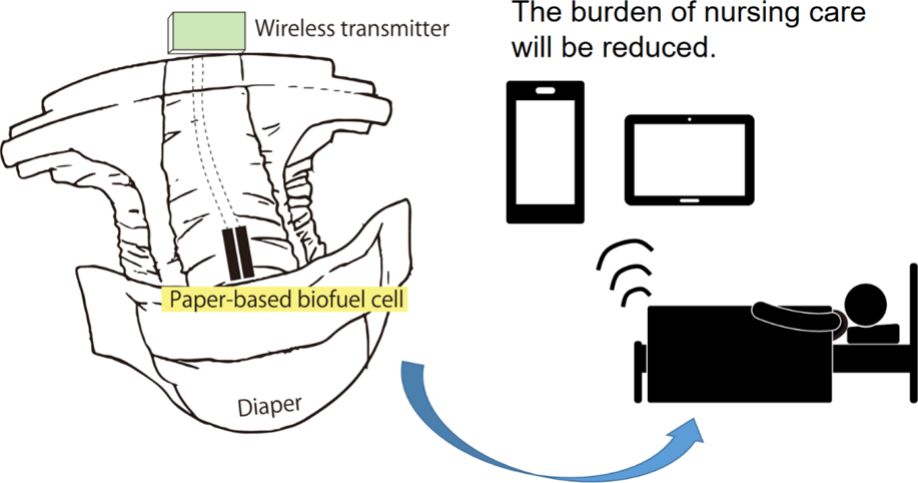

A self-driven sensor that can detect urine and urine sugar and can be mounted on diapers is desirable to reduce the burden of long-term care. In this study, we created a paper-based glucose biofuel cell that can be mounted on diapers to detect urine sugar. Electrodes for biofuel cells were produced by printing MgO-templated porous carbon on which poly(glycidyl methacrylate) was modified using graft polymerization. A new bioanode was prepared through covalently modifying flavin-adenine-dinucleotide-dependent glucose dehydrogenase and azure A with pendant glycidyl groups of poly(glycidyl methacrylate). We prepared a cathode with covalently bonded bilirubin oxidase. Covalent bonding of enzymes and mediators to both the bioanode and biocathode suppressed elution and improved stability. The biofuel cell could achieve a maximum output density of 0.12 mW cm^–2^, and by combining it with a wireless transmission device, the concentration of glucose sensed from the transmission frequency was in the range of 0–10 mM. The sensitivity of the sensor was estimated at 0.0030 ± 0.0002 Hz mmol^–1^ dm^3^. This device is expected to be a new urine-sugar detection device, composed only of organic materials with a low environmental load and it can be useful for detecting postprandial hyperglycemia.

Self-powered glucose biosensors have attracted considerable attention.^1−4^ These react enzymatically to the glucose in a body fluid to generate electric power and use this electric power to send a signal from a transmitter.^5−7^ The power generated by a biosensor using glucose as fuel is dependent on the glucose concentration, and it is possible to measure the glucose concentration from the power value. Contact lens-type sensors that monitor glucose in tears, as self-powered glucose sensors,^8,9^ and those that monitor glucose in saliva^10,11^ have been developed. These are expected to be used as blood glucose-monitoring tools for patients with diabetes.

However, monitoring glucose contained in urine (urinary sugar) is also very important. Urine sugar is closely related to blood glucose,^12,13^ and it is detected when the blood glucose level exceeds the threshold that can be processed by the kidney. If urine sugar is detected, timely examination at a medical institution is required. In addition, even if the fasting urine sugar level is within the normal range, postprandial hyperglycemia occurs in which the postprandial blood glucose level rises and the urinary sugar level also increases. Postprandial hyperglycemia is seen in the early stages of diabetes, which often progresses to severe diabetes. Therefore, it is important to confirm the change in urine sugar levels in the diabetes test. In patients requiring long-term care, detecting urine glucose and monitoring urinary sugar levels will help prevent diabetes. In addition, at a nursing care site, hundreds of diapers need to be checked regularly for the presence of urine. This is an extremely difficult task, and if the presence or absence of urine glucose can be detected using a device equipped with a biofuel cell that can be embedded in the diaper, the burden of nursing care will be reduced. Since the measurement solution is urine, there is no need for collecting blood and therefore, reducing the stress on the caregiver. This is expected to be useful for home care.

We focused on an enzymatic biofuel cell that generates electricity from urine glucose.^14−16^ We previously reported a paper-based glucose biofuel cell fabricated using screen printing^17,18^ for a self-powered urine glucose biosensor. Biofuel cells do not require a separator because of the substrate selectivity of the enzyme and take advantage of the fact that they can be freely designed, as compared to conventional fuel cells, and utilize printing technology to create a series–parallel structure.^16,17^ Using a 5-series glucose biofuel cell array with an open circuit voltage of >3 V, a buzzer can be sounded and the LED can be turned on without any other booster circuit.^17^ In addition, the glucose concentration in artificial urine was determined, and it was confirmed that there was almost no inhibition of enzyme activity by the components in artificial urine.

However, when mounting on diapers, a paper-based biofuel cell should be small and driven by a small amount of urine. For arrays, in cases where urine is not sufficiently distributed, in other words, patients with low urine volumes, setting up the cell is challenging. Even if it is disposable, the improvement of drive stability during power generation by the biofuel cell is still an important parameter for diaper-type biofuel cells. Several researchers achieved to operate wireless transmitters using biofuel cells combining a supercapacitor. For example, Monsalve et al. reported a hydrogen/oxygen biofuel cell that was able to power a wireless transmission system.^19^ Sode et al. proposed a bio-capacitor combining a capacitor with a biofuel cell.^5^ The power generated by the biofuel cell is stored in a capacitor and the information is transmitted at regular intervals using a wireless transmission system.

However, there are no earlier reports of diapers equipped with biofuel cells using a step-up wireless transmission circuit. Therefore, in this study, we attempted to create a new self-powered diaper biosensor that combines a low-power wireless transmission device and a paper substrate glucose biofuel cell (single cell).

To improve the stability of the paper-based biofuel cell we developed earlier,^14^ we prepared a bioanode in which azure A and flavin-adenine-dinucleotide-dependent glucose dehydrogenase (FAD-GDH) were immobilized by covalent bonding on porous carbon, namely, MgO-templated mesoporous carbon (MgOC),^20−23^ using electron beam-induced graft polymerization. Azure A is known as a mediator of the glucose biosensor.^24^ Using the graft polymerization technique, poly(glycidyl methacrylate) (PGMA) was induced on the MgOC surface. The PGMA has epoxy groups that react with amine groups of FAD-GDH and azure A. Therefore, the enzyme was covalently bound to the graft-polymerized MgO-templated carbon (GMgOC) through the GMA polymers. Previously, we reported stable immobilization of FAD-GDH^25^ and lactate oxidase^23^ on GMgOC. Amino-ferrocenes can also be immobilized on GMgOC.^22^ However, to the best of our knowledge, GMgOC has never been subjected to paper-based biofuel cells. We also prepared a biocathode in which bilirubin oxidase (BOD) was immobilized on GMgOC. In addition, a biofuel cell was manufactured using the prepared bioanode and biocathode, mounted on a diaper, and evaluated for wireless transmission.

## Experimental Section

### Materials

MgOC (average pore diameter: 100 nm, MJ (3)100) was purchased from Toyo Tanso (Japan). GMA, *N,N*-dimethylformamide (DMF), 1-methylpyrrolidin-2-one (NMP), and acetonitrile were obtained from Wako Pure Chemical Industries (Japan). FAD-GDH (205 U mg^–1^) was purchased from Amano Enzyme (Japan). Bilirubin oxidase BOD (from *Myrothecium verrucaria*, 2 U mg^–1^) was purchased from Amano Enzyme (BO “Amano3”, Japan). Azure A (Sigma-Aldrich, Japan) was used as the mediator and was dissolved in methanol. Polyvinylidene difluoride hexafluoropropylene copolymer (PVdF; KF polymer L#9305, 5% in NMP) was purchased from Kureha Corporation (Japan). All chemicals were of analytical grade. Carbon ink (JELCON CH-8) was obtained from Jujo Chemical (Japan). Filter paper (No. 1002-110) was obtained from Whatman (Japan).

### Graft Polymerization of MgO-Templated Carbon

**Figure 1:**
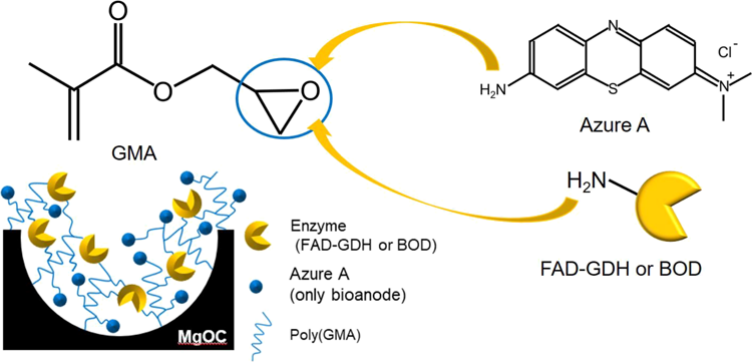
Schematic diagram of MgO-templated mesoporous carbon (MgOC) modified with poly(glycidyl methacrylate) (PGMA), namely, GMgOC, to immobilize flavin-adenine-dinucleotide-dependent glucose dehydrogenase (FAD-GDH) and azure A.

The preparation scheme of GMgOC for immobilizing FAD-GDH and azure A is provided (Figure 1). GMgOC was prepared according to a previously reported method.^26^ MgOC (4.0 g) was subjected to electron beam irradiation and was prepared in DMF with a GMA concentration of 20 vol % at 100 °C. The graft ratio of GMgOC was analyzed through thermal gravimetric analysis (TGA; DSC1 Star System, Mettler-Toledo, Japan).

### Fabrication of Bioanode, Biocathode, and Biofuel Cell

**Figure 2:**
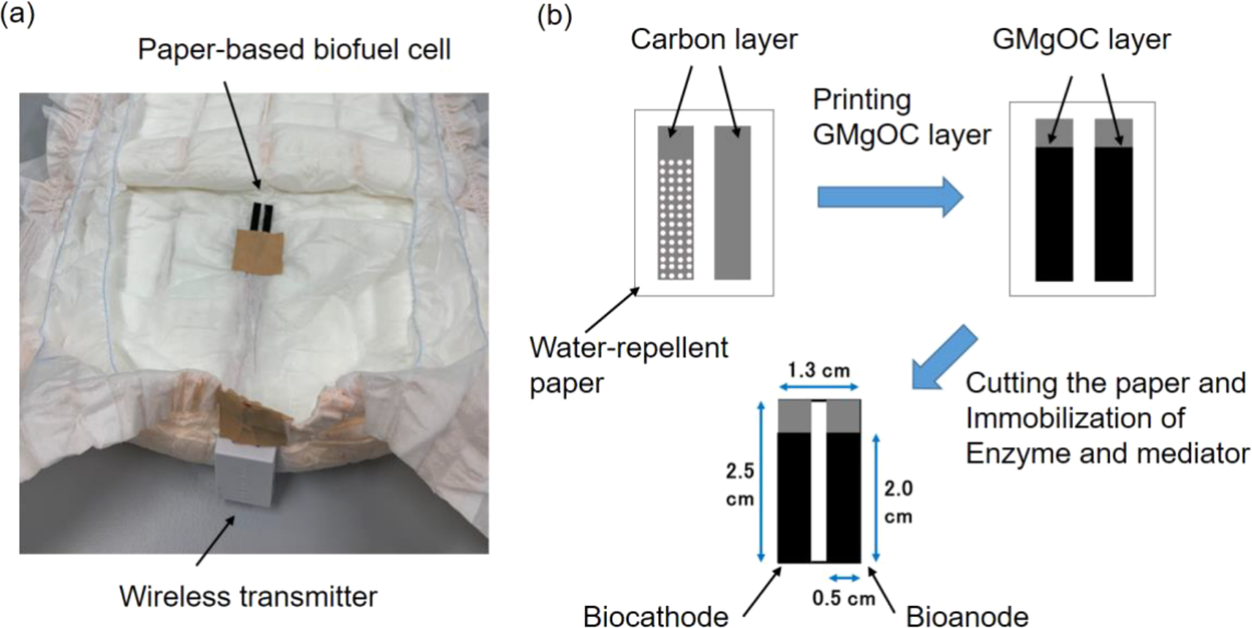
(a) Photograph of the paper-based diaper biofuel cell with a wireless transmitter. (b) Schematic illustration of the preparation process of the paper-based diaper biofuel cell.

The cathode was designed with a hole to supply oxygen from the air. The substrate was a filter paper (ADVANTEC No. 5A, 185 μm thick, Japan). A semi-automatic screen-printing machine (LS-150TV, Newlong Seimitsu Kogyo, Japan) was used to print the current collector wiring (lead part) on a paper substrate using carbon paste. After printing, the carbon paste was dried at 120 °C for 24 h. After that, the back of the current collector wiring was treated using a water-repellent agent (Fluorosurf FG-3030C-30, Fluorotechnology, Japan) to prevent short circuits. The porous carbon electrodes were prepared by printing porous carbon paste. The porous carbon paste was prepared by mixing 2 g of porous carbon (MgOC or GMgOC), 10 mL of PVdF, and 5 mL of NMP and kneading at 2000 rpm for 1 min. After printing the prepared porous carbon paste, the electrodes were dried at 60 °C. The apparent surface area of the porous carbon electrode was 1 cm^2^ (0.5 × 2.0 cm) (Figure 2a,b).

Before enzyme modification, UV ozone treatment for 15 min was performed to remove impurities on the electrode surface as a preliminary preparation. Thereafter, to prepare a bioanode, 20 μL of azure A was dropped and dried for 30 min at 25 °C under reduced pressure conditions. Twenty microliters of FAD-GDH (20 U/μL) solution, in which FAD-GDH was dissolved in 10 mM phosphate buffer solution (pH 7.0) containing 0.01% surfactant triton X-100 (Roche Diagnostics GmbH, Switzerland), was dropped and the bioanode was dried under reduced pressure for 1 h. To prepare the biocathode, 20 μL of BOD (1 U/μL) dissolved in 10 mM phosphate buffer solution (pH 7.0) containing 0.01% triton X-100 was dropped onto the electrode surface. The biocathode was then dried under reduced pressure for 1 h.

### Electrochemical Measurement

The performance of the bioanode and biocathode was analyzed by cyclic voltammetry using a potentio/galvanostat (PalmSens, Emstat Blue, the Netherlands) with a three-electrode method. An Ag/AgCl/saturated KCl electrode and a Pt wire were used as the reference and counter electrodes, respectively. The bioanode and biocathode were dipped in 1.0 mol dm^–3^ phosphate buffer (pH 7.0) containing 0–0.1 mol dm^–3^ glucose.

All electrochemical measurements were performed at 37 °C. The current density and power density were calculated based on the projected surface area of the electrodes. All measurements were performed in triplicate. Error bars were determined using a Student’s *t* distribution at 90.0% confidence level (*n* = 3). The wireless transmission experiment was conducted by connecting a wireless transmitter (CLEAN-boost, ABLIC, Japan) and a biofuel cell.

## Results and Discussion

### Immobilization of FAD-GDH on Paper-Based GMgOC Electrode

**Figure 3:**
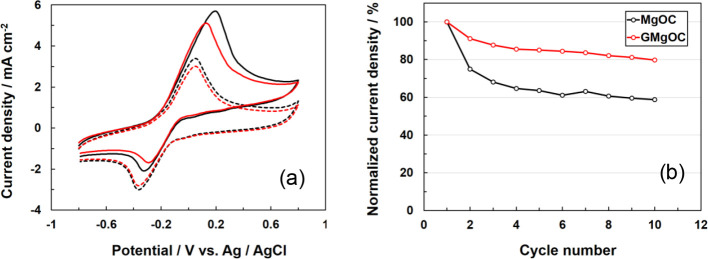
(a) Cyclic voltammograms of the bioanode modified with FAD-GDH/azure A in 1 mol dm^–3^ phosphate buffer in the presence of 100 mmol dm^–3^ glucose using MgOC (solid black curve) and GMgOC (solid red curve) and in the absence of glucose using MgOC (dashed black curve) and GMgOC (dashed red curve). Scan rate: 10 mV s^–1^. (b) Changes in normalized current density of the FAD-GDH/azure A-immobilized GMgOC electrode (red circle) and FAD-GDH/azure A-immobilized MgOC electrode (black circle), as estimated from the cyclic voltammograms (10 cycles) obtained in 1 mol dm^–3^ phosphate buffer solution containing 100 mmol dm^–3^ glucose at +0.3 V.

The success of the graft polymerization of GMA on the MgOC surface was confirmed by FT-IR spectroscopy, as previously reported by us.^22^ A grafting rate of 10.2% was determined by TGA. The cyclic voltammograms of the bioanodes using MgOC and GMgOC in the presence of 100 mmol dm^–3^ glucose and in the absence of glucose are shown (Figure 3a). Two redox peaks were observed at −0.3 and +0.05 V in the absence of glucose (black and red dashed curves).

A clear increase in the catalytic current was observed at each electrode using MgOC and GMgOC (black and red dashed curves) in the presence of 100 mmol dm^–3^ glucose. The catalyst current values were 5.7 and 5.2 mA cm^–2^, respectively, indicating that the performance was almost the same. The mediator, azureA, was cast on the electrode in sufficient amount to react with FAD-GDH. The initial immobilization amounts of FAD-GDH and azure A were the same for MgOC and GMgOC. Therefore, the first cycle values of both noncatalytic current and catalytic current were almost equal for MgOC and GMgOC. A plot of the current value at +0.3 V after repeated cyclic voltammetry for 10 cycles is shown (Figure 3b). The current value in the first cycle was normalized to 100%. After 10 cycles, the current was maintained at 59% for the MgOC electrode. In contrast, the GMgOC electrode maintained an 80% current value.

The relationship between the current value and the number of cycles when only azure A was immobilized on GMgOC is shown (Figure S1). After 10 cycles, the current decreased to 49% for the MgOC electrode. However, the GMgOC electrode maintained an 82% current value. These results indicate that azure A and FAD-GDH were stably immobilized on the MgOC surface via reactions with PGMA. The decrease in the current value in the second cycle at the GMgOC electrode was attributed to the elution of unfixed azure A and FAD-GDH. However, it can be seen that the GMgOC-based bioanode was very stable after the third cycle. The FAD-GDH-immobilized bioanode was more stable than the bioanode using MgOC, because the oxidized azure A on the electrode surface was reduced with FAD-GDH, and the elution was suppressed.

### Evaluation of Enzyme Immobilization in Biocathode

**Figure 4:**
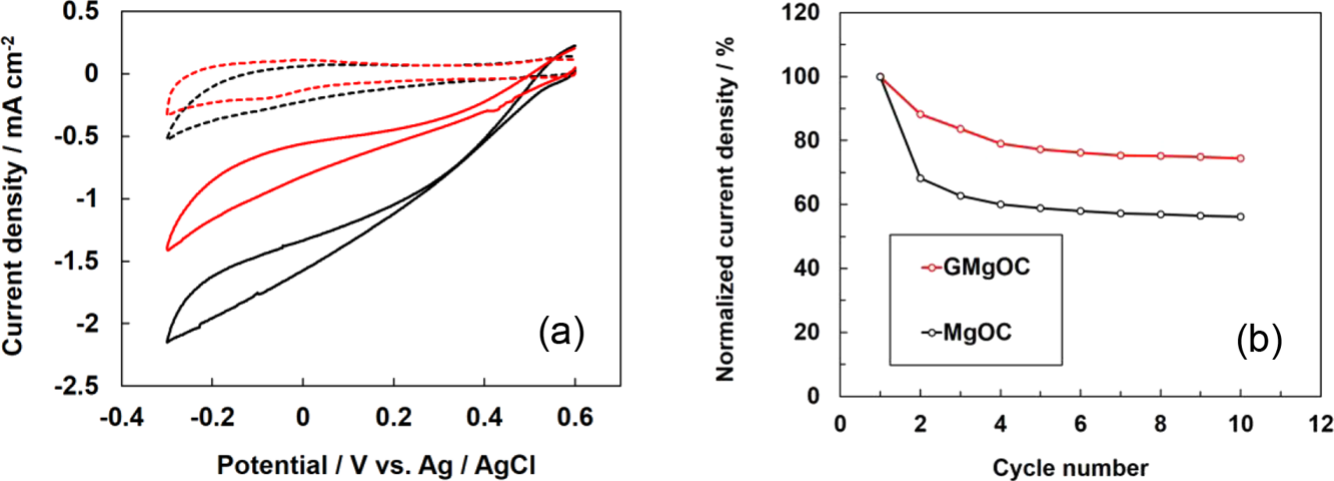
(a) Cyclic voltammograms of the biocathode modified with BOD in 1 mol L^–1^ phosphate buffer using MgOC (solid black curve) and GMgOC (solid red curve) and the biocathode unmodified with BOD using MgOC (dashed black curve) and GMgOC (dashed red curve). Scan rate: 10 mV s^–1^. (b) Changes in normalized current density of the BOD-immobilized GMgOC electrode (red circle) and BOD-immobilized MgOC electrode (black circle), as estimated from the cyclic voltammograms (10 cycles) obtained in 1 mol L^–1^ phosphate buffer solution at −0.3 V.

The cyclic voltammetry of the biocathode using MgOC and GMgOC is shown (Figure 4a). An enzymatic oxygen reduction reaction of BOD appeared clearly on the biocathodes using both MgOC and GMgOC with BOD (black and red solid lines). Maximum current densities of −2.1 and – 1.4 mA cm^–2^ were obtained for the biocathode using MgOC and GMgOC, respectively. The onset potential did not differ between GMgOC and MgOC and was around 0.5 V. This value was almost the same as the previously reported value.^27^

Compared with that of the bioanode, the current density ratio of the biocathode was higher when MgOC was used as the biocathode. When GMgOC was used, BOD may have become less oriented due to the binding of PGMA with lysine residue of BOD, and the amount of BOD with electrochemical activity may have decreased. It is also possible that the electrochemical activity of the BOD was reduced due to the effect of the cross-linking on the BOD.

A plot of the current value at −0.3 V after repeating cyclic voltammetry for 10 cycles is shown (Figure 4b). The biocathode using MgOC was maintained at a current value of 56%. However, the biocathode using GMgOC maintained a current value of 74% after 10 cycles. From these results, it was confirmed that the elution of BOD was suppressed by the covalently bonding of the amino group of BOD with the epoxy group of PGMA.

### Evaluation of the Performance of the Paper-Based Biofuel Cell

**Figure 5:**
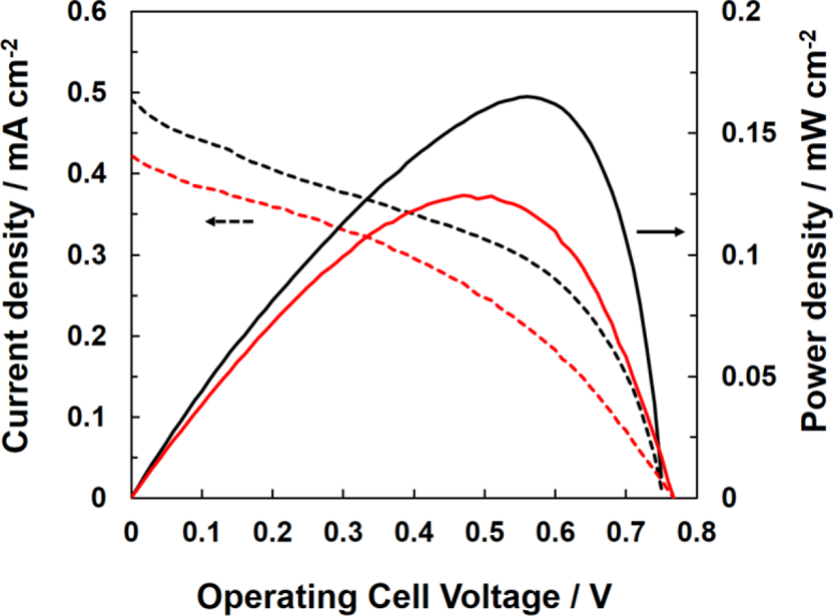
Cell voltage–current density and cell voltage–power density curves of the paper-based biofuel cell operated using MgOC (black curves) and GMgOC anodes (red curves) in 100 mmol dm^–3^ glucose.

The performance of the paper-based biofuel cells using MgOC and GMgOC in 100 mmol dm^–3^ glucose is shown (Figure 5). An open circuit voltage of 0.76 V, a maximum current density of 0.49 mA cm^–2^, and a maximum output density of 0.17 mW cm^–2^ were obtained for the biofuel cell using MgOC. However, an open circuit voltage of 0.77 V, a maximum current density of 0.42 mA cm^–2^, and a maximum output density of 0.12 cm^–2^ were obtained using GMgOC. The output of the GMgOC-based biofuel cell was lower than that of the MgOC-based biofuel cell, because of the biocathode performance.

The current density was lower when GMgOC was used, but the stability was higher when GMgOC was used, as mentioned previously. In this type of diaper biofuel cell as a self-powered biosensor, when the amount of electricity is stored above a certain level, wireless communication is performed. If the current value is not stable, the accurate concentration cannot be estimated. Therefore, both output density and a stable current value are critical.

**Figure 6:**
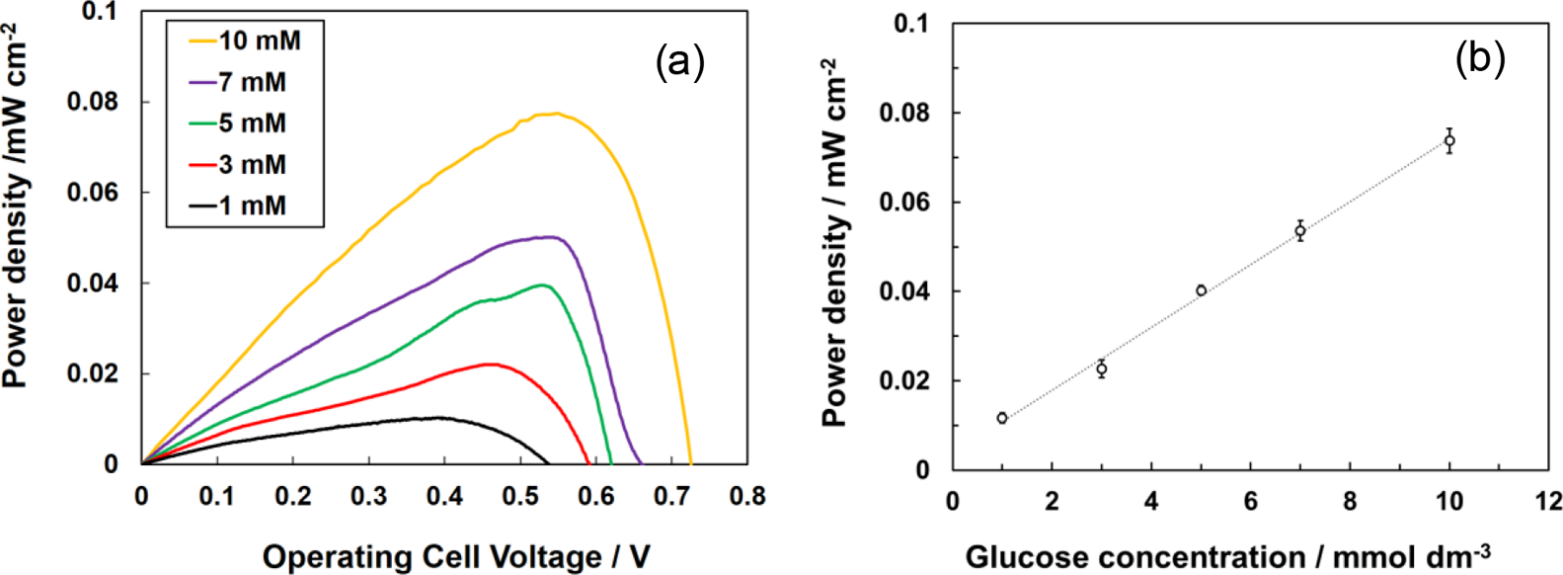
(a) Dependence of the power–current curve on the concentration of glucose using GMgOC anode. (b) Maximum power plotted against glucose concentration. Error bars were determined using Student’s *t* distribution at 90.0% confidence level (*n* = 3).

The relationship between the glucose concentration and output power density of the paper-based biofuel cell was evaluated at different glucose concentrations (1, 3, 5, 7, and 10 mmol dm^–3^) (Figure 6). The output of the biofuel cell showed a linear dependence on the glucose concentration. These results suggest that the biofuel cell can be used as a self-driven urinary glucose sensor. The sensitivity of the sensor was estimated at 0.0071 ± 0.0002 mW cm^–2^ mmol^–1^ dm^3^. The value of the coefficient of determination (*R*^2^) was 0.9927. The 90% confidence intervals indicate that the response of the sensor was highly reproducible. The maximum error was ±3.7% at 10 mmol dm^–3^.

### Wireless Transmission Test for Paper-Based Biofuel Cell as a Self-Powered Urine Sugar Biosensor

A wireless transmission test was performed using a Bluetooth Low Energy wireless transmitter (CLEAN-Boost, ABLIC, Japan). The operating principle is that the power generated by the biofuel cell is stored in a capacitor, boosted, and released when a certain amount of energy is obtained. The interval time is the time until the power is stored in the capacitor, which depends on the output of the biofuel cell and has the following relationship:
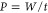
1where *P* is the power (W), *W* is the energy that can be stored in the capacitor (J), and *t* is the interval time (s). Therefore, if there is a correlation between the frequency, which is the reciprocal of the transmission interval [= transmission frequency (Hz)], and the output of the biofuel cell, the glucose concentration can be measured from the transmission frequency.

**Figure 7:**
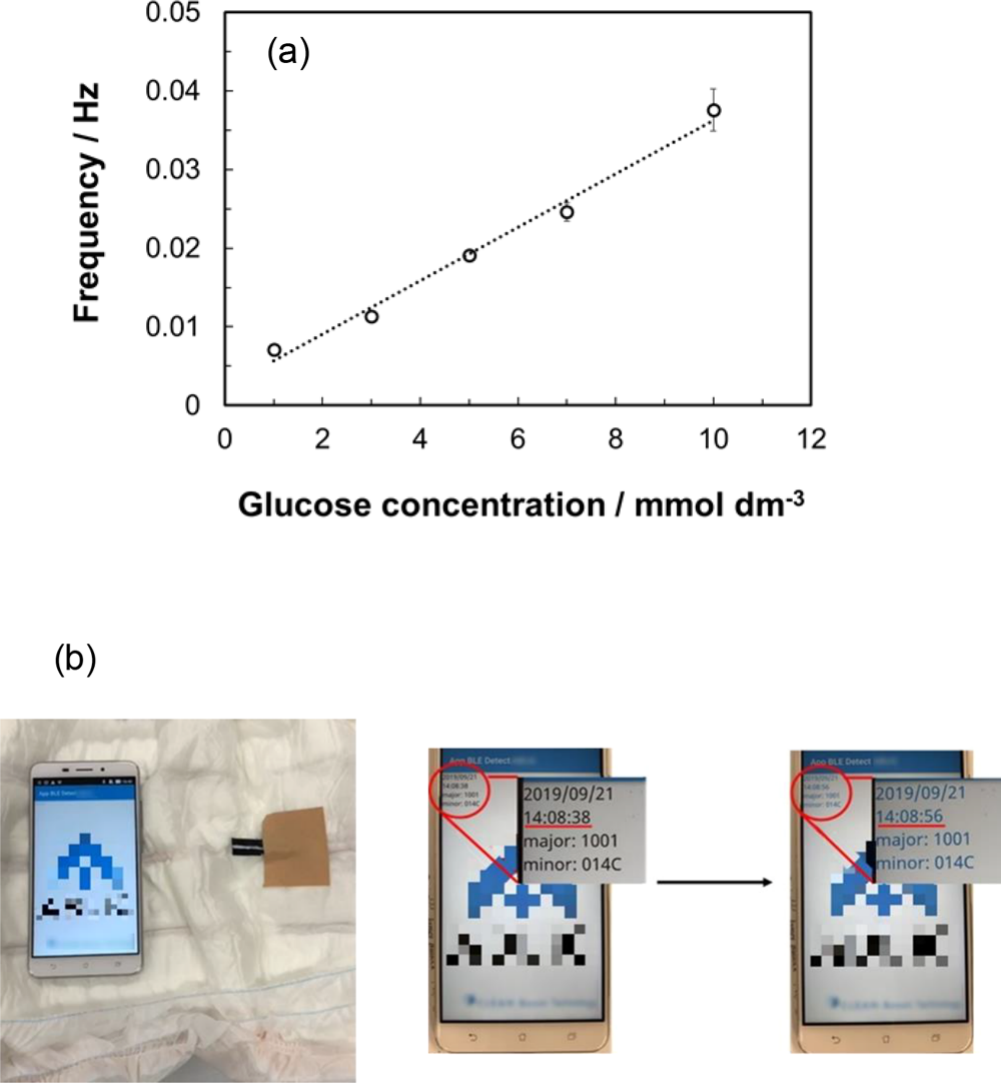
(a) Correlation between transmission frequency and glucose concentration on the paper-based diaper biofuel cell. (b) Photograph of the wireless transmission test in 10 mmol dm^–3^ glucose. Error bars were determined using a Student’s *t* distribution at 90.0% confidence level (*n* = 3). The company logos are hidden by mosaic.

In the case of a 100 mmol dm^–3^ glucose solution, the interval time was within 1 s and the smartphone flashed at the interval time (SI movie). Figure 7 shows the relationship between the glucose concentration and transmission frequency in the biofuel cell. A linear relationship was obtained at 1–10 mmol dm^–3^ glucose. The sensitivity of the sensor was estimated at 0.0030 ± 0.0002 Hz mmol^–1^ dm^3^. The value of the coefficient of determination (*R*^2^) was 0.9920. The 90% confidence intervals indicate that the responses of the sensor were highly reproducible. The maximum error was ±7% at 10 mmol dm^–3^. From this result, it can be seen that urine sugar can be detected in a very short time using the biofuel cell. This was achieved because we were able to produce an unprecedented high-power and stable paper-based biofuel cell. Diagnosis of diabetes is made by checking fasting blood glucose level of 126 mg/dL (7.0 mmol dm^–3^) at least twice.^28^ Since this sensor shows good linearity and reproducibility in the determination range of diabetes, it can be used for the determination of diabetes.

## Conclusions

The output that the paper-based diaper biofuel cell produced was approximately 0.12 mW at 25 mmol dm^–3^ glucose, which was sufficient to drive a power-saving wireless transmission device. The use of GMgOCs improved drive stability and the output power depended on the glucose concentration in the concentration range of 0–10 mmol dm^–3^. There was a linear relationship between the maximum output density and the glucose concentration, which can be measured by wireless transmission. In the near future, we plan to evaluate the long-term storage stability and conduct a demonstration test by mounting the biofuel cell on diapers for long-term care recipients. The concept of this study is a very promising tool toward the development of self-powered wearable biosensors.

## Supporting Information Available

Cyclic voltammogram of the bioanode modified with azure A (PDF)
Movie of the wireless transmission test (MP4)

## Abbreviations

FAD-GDH: flavin-adenine-dinucleotide-dependent glucose dehydrogenase
MgOC: MgO-templated mesoporous carbon
PGMA: poly(glycidyl methacrylate)
GMgOC: graft-polymerized MgO-templated carbon
BOD: bilirubin oxidase
DMF: *N,N*-dimethylformamide
NMP: 1-methylpyrrolidin-2-one
PVdF: polyvinylidene difluoride hexafluoropropylene copolymer
TGA: thermal gravimetric analysis.
