# Supplementary material for: Self-Powered Diaper Sensor with Wireless Transmitter Powered by Paper-Based Biofuel Cell with Urine Glucose as Fuel
Source: ACS Sens. 2021 Jul 15;6(9):3409–15. doi: 10.1021/acssensors.1c01266 (PMC8477385; doi:10.1021/acssensors.1c01266)
Supplement: Supplementary file 1 — se1c01266_si_001.pdf [file se1c01266_si_001.pdf]

Supporting Information

Self-powered Diaper Sensor with Wireless Transmitter Powered by Paper-based Biofuel Cell with Urine Glucose as Fuel

Isao Shitanda[†,‡,*], *Yuki Fujimura*[†], *Tatsuya Takarada*[†], *Ryo Suzuki*[†], *Tatsuo Aikawa*[†], *Masayuki Itagaki*[†,‡], *and Seiya Tsujimura*[‡,§]

[†]Department of Pure and Applied Chemistry, Faculty of Science and Technology, Tokyo University of Science, 2641, Yamazaki, Noda, Chiba 278-8510, Japan

[‡]Research Institute for Science and Technology, Tokyo University of Science, 2641 Yamazaki, Noda, Chiba 278-8510, Japan

[§]Division of Material Science, Faculty of Pure and Applied Science, University of Tsukuba, 1-1-1, Tennodai, Tsukuba, Ibaraki 305-5358, Japan

Cyclic voltammogram of the bioanode modified with Azure A

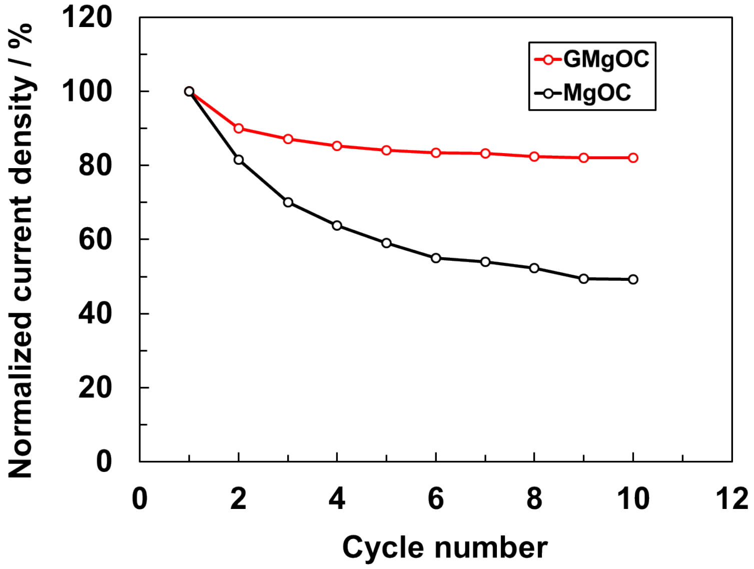

Figure S1 Cyclic voltammograms of the bioanode modified with Azure A in 1 mol dm^{-3}

phosphate buffer using MgOC (solid black curve) and GMgOC (solid red curve).

Movie of the wireless transmission test

SI.mp4
